# A Multicenter Retrospective Analysis of the Utility of Intravascular Lithotripsy in Underexpanded Stents

**DOI:** 10.1016/j.jscai.2025.103600

**Published:** 2025-05-02

**Authors:** Lance Ng, Bernard Wong, Seif El-Jack, Wil Harrison, Mark Webster, Jithendra Somaratne

**Affiliations:** a Auckland City Hospital, Auckland, New Zealand; b Now with Cardiac Catheterisation Laboratory, Peter Munk Cardiac Center, Toronto General Hospital, Toronto, Ontario, Canada; c North Shore Hospital, Auckland, New Zealand; d Middlemore Hospital, Auckland, New Zealand

**Keywords:** calcium modification, in-stent restenosis, intravascular lithotripsy, stent underexpansion

## Abstract

**Background:**

Stent underexpansion is a key determinant to both short- and long-term outcomes after percutaneous coronary intervention (PCI). Current strategies available have inherent limitations in the setting of stent underexpansion, and intravascular lithotripsy (IVL) remains off-label for in-stent use. Our study aimed to demonstrate the safety and efficacy of IVL use in underexpanded stents.

**Methods:**

We undertook a retrospective analysis of PCIs involving IVL at 3 centers in New Zealand between September 2018 and November 2023. We identified cases in which IVL was utilized for both old and new in-stent lesions. The primary outcome was a 12-month major adverse cardiac events (cardiac death, nonfatal myocardial infarction [MI], or ischemia-driven target vessel revascularization [ID-TVR]). Secondary outcomes were procedural success (<30% residual stenosis), 30-day cardiac and noncardiac death, nonfatal MI, ID-TVR, and stent thrombosis. Angiographic and intravascular imaging outcomes were also analyzed.

**Results:**

Between September 2018 and November 2023, 68 of 743 IVL cases involved in-stent lesions. Of the cases, 69% were acute coronary syndrome presentations. Twelve-month major adverse cardiac events were 8.8%. Procedural success was 87%. At 30 days, there was 1 noncardiac death but no cardiac death, nonfatal MI, ID-TVR, or stent thrombosis events. Serious complications included 2 cases of slow flow. Angiographic mean minimal lumen diameter pre-PCI was 0.89 ± 0.54 mm, post-IVL was 2.40 ± 0.60 mm, and post-stenting was 3.01 ± 0.69 mm. Intravascular imaging use was 41%; mean minimal lumen area was 3.60 ±1.78 mm^2^ pre-PCI and 8.71 ± 3.28 mm^2^ post-PCI.

**Conclusions:**

Our multicenter retrospective analysis demonstrates that IVL is a safe and effective tool in the treatment of underexpanded stents with 12-month MACE rates comparable to those of de novo coronary lesions and a high rate of procedural success. Larger, randomized studies are required to elucidate the optimal approach for treating underexpanded stents.

## Introduction

Stent underexpansion is a key determinant to both short- and long-term outcomes after percutaneous coronary intervention (PCI).^1^, ^2^, ^3^, ^4^, ^5^ Coronary artery calcification is the major cause of stent underexpansion. Considering the increasing burden of calcific coronary disease being treated, which is a function of both population aging as well as growing capabilities and expectation with percutaneous therapies,^6^ understanding the interventional tools available to treat stent underexpansion is critical.

Intravascular lithotripsy (IVL) is now a well-established therapy, which utilizes high-frequency pulses of ultrasound (acoustic pressure waves lasting 1 ms are delivered at a rate of 1 pulse per second) to create cracks in both superficial and deep calcium in the walls of the coronary artery, thus allowing optimal stent expansion when treating calcific coronary lesions.^7^ However, its use within both newly implanted and older preexisting stents has not been evaluated in a randomized setting.^8^^,^^9^ A number of small registries evaluate minimal stent area and short-term (in-hospital and 30-day) outcomes after IVL use in underexpanded stents.^10^, ^11^, ^12^ We sought to evaluate the procedural success and 12-month outcomes of using IVL as a therapy in underexpanded stents because the alternative options currently utilized have their own significant limitations. High-pressure balloon inflations (with noncompliant or OPN balloons [SIS Medical]) and modified (scoring or cutting) balloons all carry a risk of barotrauma and perforation. Atherectomy devices (namely rotational; orbital is currently contraindicated within previous stents^13^^,^^14^) and excimer laser coronary angioplasty (ELCA) carry the risk of slow flow or no reflow, perforation, and flow-limiting iatrogenic coronary dissection.^15^

## Methods

We undertook a retrospective analysis of all PCIs utilizing IVL with the Shockwave IVL Catheter (Shockwave Medical) between September 2018 and November 2023 at 3 centers in New Zealand and identified all cases in which IVL was utilized for in-stent lesions, including both newly deployed and previously deployed stents.

The primary outcome was a 12-month major adverse cardiac events (MACEs: cardiac death, nonfatal myocardial infarction [MI], or ischemia-driven target vessel revascularization [ID-TVR]). Secondary outcomes included procedural success, defined as <30% residual stenosis at the conclusion of the procedure, 30-day cardiac and noncardiac death, nonfatal MI, ID-TVR, and stent thrombosis at 30 days. Angiographic and intravascular imaging outcomes were also analyzed. Quantitative coronary angiography and intravascular ultrasound measurements were performed independently by the authors post procedure. Ultreon 1.0 (Abbott) was used for optical coherence tomography analysis.

Continuous data are expressed as mean ± SD and categorical variables are expressed as frequencies and percentages. Statistical analyses were performed using Stats.Blue software.

Ethical approval for a retrospective analysis was obtained through the local hospital network research office and ethics committee.

## Results

Between September 2018 and November 2023, there was a total of 734 PCIs for which IVL was utilized across 3 centers in New Zealand. Of the 734 PCIs utilizing IVL, 68 of 734 (9.3%) were for in-stent lesions (both newly deployed and preexisting stents). Follow-up details were available for all 68 cases including outcomes to 12 months.

Our population had a mean age of 69 years and were predominantly male (72%). Diabetics made up 44% of the cohort, and a significant proportion (40%) had impaired renal function (estimated glomerular filtration rate <60 mL/min/1.73 m^2^). Over two-thirds of the lesions occurred in acute coronary syndrome presentations (69%). Detailed baseline clinical characteristics are provided in Table 1.

**Table 1:** Baseline characteristics.

Characteristics	N = 68
Age, y	68.9 ± 8.4
Male sex	49 (72.0)
Diabetes	30 (44.1)
Hypertension	45 (66.2)
Hyperlipidemia	38 (55.9)
Prior myocardial infarction	38 (55.9)
Prior coronary artery bypass grafting	13 (19.1)
Prior stroke or transient ischemic attack	6 (8.8)
Current smoker	4 (5.9)
Renal insufficiency (eGFR <60 mL/min/1.73 m^2^)	27 (39.7)
LVEF <40%^a^	14 (21.2)
Acute coronary syndrome	
STEMI	5 (7.6)
NSTEMI	30 (44.1)
Unstable angina	12 (17.6)
Angina score (if stable coronary disease or staged)	
Class 0	2 (2.9)
Class I	5 (7.6)
Class II	8 (11.8)
Class III	6 (8.8)
Class IV	0 (0)

Values are mean ± SD or n (%).

eGFR, estimated glomerular filtration rate; LVEF, left ventricular ejection fraction; NSTEMI, non-ST-elevation myocardial infarction; STEMI, ST-elevation myocardial infarction.

a N = 66. 2 patients did not have LVEF measured or did not have LVEF results available.

Over half of these underexpanded stent lesions occurred in stents implanted >1 year prior to the PCI of the underexpanded stent using IVL. IVL was used in stents <1 year old in 25% of the cohort, and 13% had IVL performed in a newly-implanted stent (during the same procedure as a bail-out, after the stent had been deployed and was found to be underexpanded).

The majority of these lesions (77%) were subsequently stented with a late-generation drug-eluting stent following IVL, but 15% had IVL and then a drug-coated balloon (rather than a drug-eluting stent) for the underexpanded stent. Rotational atherectomy was utilized in only 1 case (1.5%). The mean minimal lumen diameter pre-PCI was 0.89 ± 0.54 mm, post-IVL was 2.40 ± 0.60 mm, and after stenting (if stented) was 3.01 ± 0.69 mm. Intravascular imaging was used in 41% of cases in which the mean minimal lumen area was 3.60 ± 1.78 mm^2^ pre-PCI and 8.71 ± 3.28 mm^2^ post-PCI. Stent expansion of >80% was achieved on intravascular imaging in 82% of those who had pre-PCI and post-PCI imaging performed. Comprehensive details regarding the procedural characteristics are provided in Table 2.

**Table 2:** Procedural characteristics.

Characteristics	N = 68
Basic laboratory data	
Contrast volume, mL	149.1 ± 77.8
Access	
Radial	58 (85.3)
Femoral	10 (14.7)
Lesion characteristics and treatment	
Underexpanded stent (target lesion) age	
Newly deployed	9 (13.2)
<1 y	16 (23.5)
>1 y^a^	43 (63.2)
Maximum predilatation balloon size, mm	3.34 ± 0.54
Mean IVL balloon size, mm	3.51 ± 0.43
Concurrent rotational atherectomy	1 (1.5)
Device delivered	
Stent	52 (76.5)
Drug-coated balloon	13 (15.1)
None	3 (4.4)
No. of stents implanted (of 52 stented)	
1	27 (51.9)
2	22 (42.3)
3	3 (5.8)
≥4	0 (0)
Total stent length, mm	45.1 ± 23.0
Poststent dilatation (of 52 stented)	52 (100)
Angiographic characteristics and outcomes	
Target vessel	
Left main stem	6 (8.8)
Left anterior descending	20 (29.4)
Left circumflex	11 (16.2)
Right coronary artery	31 (45.6)
Bifurcation with side branch involvement	9 (13.2)
Reference vessel size, mm	3.47 ± 0.52
Lesion length, mm	16.2 ± 9.2
Pre-PCI minimal lumen diameter, mm	0.89 ± 0.54
Pre-PCI diameter stenosis, %	74.9 ± 14.8
Post-IVL minimal lumen diameter, mm	2.40 ± 0.60
Post-IVL diameter stenosis, %	30.4 ± 16.2
Post-IVL acute gain, mm	1.49 ± 0.66
Poststent minimal lumen diameter, mm	3.01 ± 0.69
Poststent stenosis <30%	59 (86.8)
Poststent acute gain, mm	2.13 ± 0.81
Intravascular imaging characteristics and outcomes	
Modality used	
Intravascular ultrasound	20 (29.4)
Optical coherence tomography	8 (11.8)
None	40 (58.8)
Pre-PCI minimal luminal area, mm^2^	3.60 ± 1.78
Calcium distribution (of 28 who had IVUS/OCT)	
360°	13 (46.4)
270°-360°	5 (17.9)
<270°	2 (7.1)
Nodular	8 (28.6)
Post-PCI minimal stent area, mm^2^	8.71 ± 3.28
Stent expansion >80% (compared to mean reference lumen area^b^)	18 (81.8)^c^
Serious angiographic complications	
Flow-limiting dissection	0 (0)
Perforation	0 (0)
Abrupt closure	0 (0)
Slow flow/no reflow	2 (7.1)

Values are mean ± SD or n (%).

IVL, intravascular lithotripsy; PCI, percutaneous coronary intervention.

a All in-stent lesions had a single layer of pre-exiting (new or old) stent—there were no lesions treated with ≥2 layers of stent.

b Mean reference lumen area calculated as the sum of proximal and distal reference lumens divided by 2.

c Only 22 of the 28 who had IVUS or OCT pre-PCI had post-PCI imaging performed.

The primary outcome of 12-month MACE occurred in 8.8% of the cohort, comprised of cardiac death (2.9%), nonfatal MI (4.4%), and ID-TVR (1.5%). Procedural success (<30% residual stenosis) occurred in 87% of cases. There was a single noncardiac death within the first 30 days. The only serious procedural complications to occur were 2 cases of slow-flow post-IVL. There were no cases of flow-limiting dissection, perforation, or abrupt vessel closure. The primary and secondary end points are summarized in Table 3 and Central Illustration.

**Table 3:** Primary and secondary end points.

End points	N = 68
Primary end points	
12-mo MACE	6 (8.8)
Cardiac death	2 (2.9)
Nonfatal myocardial infarction	3 (4.4)
Ischemia-driven target vessel revascularization	1 (1.5)
Secondary end points	
Procedural success (residual stenosis <30% without in-hospital MACE)	59 (86.8)
Cardiac death at 30 d	0 (0)
Noncardiac death at 30 d	1 (1.5)
Target lesion failure at 30 d	0 (0)
Stent thrombosis at 30 d	0 (0)
12-mo MACE by presentation	
Acute coronary syndrome	4/47 (8.5)^a^
Stable coronary disease	2/21 (9.5)^b^

Values are n (%)

MACE, major adverse cardiac event.

a MACE for acute coronary syndromes was calculated using the denominator of 47 (total number of acute coronary syndromes).

b MACE for stable coronary disease was calculated using the denominator of 21 (total number of stable coronary disease patients).

**Central Illustration:**
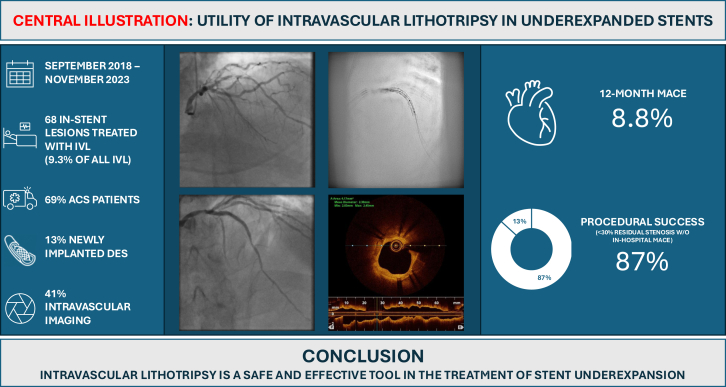
**Utility of intravascular lithotripsy in underexpanded stents.** ACS, acute coronary syndrome; DES, drug-eluting stent; IVL, intravascular lithotripsy; MACE, major adverse cardiac event; w/o, without.

## Discussion

Our multicenter retrospective analysis demonstrates that IVL is a safe and effective tool for treating both newly deployed as well as older underexpanded stents. The observed 12-month MACE rate of 8.8% is in keeping with rates seen in large practice-based datasets for 12-month MACE post-PCI, particularly given the predominance of acute coronary syndrome presentations (69%) in our cohort.^16^, ^17^, ^18^ Equally, the rate of intraprocedural complications was low and in all cases, the IVL device was able to cross the lesion to deliver therapies (rotational atherectomy was only utilized as an additional calcium modification strategy in 1 case).

That being said, a key finding is that only 87% of cases met criteria for procedural success (residual stenosis <30%). This is significantly lower than the 90% to 95% procedural success rates for IVL use in de novo lesions seen in both the Disrupt CAD series and various registries.^7^^,^^8^^,^^19^, ^20^, ^21^ It highlights that despite best efforts with both IVL and (typically high-pressure) noncompliant balloon inflation, correcting stent underexpansion and modifying calcium sitting behind a stent structure is challenging, and such an approach is less efficacious compared to its use in de novo disease. It further doubles down on the importance and growing emphasis on identifying and modifying severe calcification up front to avoid having to use bail-out techniques in the first place, and this in turn strengthens the argument (which a burgeoning body of evidence now supports) for the use of intravascular imaging guidance in PCI of complex and calcified lesions to avoid the phenomenon of “stent regret.”^22^, ^23^, ^24^

Our cohort is a typical representation of a New Zealand population with coronary artery disease with a male preponderance, a mean age in the late 60s, and a significant diabetic population. In addition, a significant proportion of patients (40%) had impaired renal function (estimated glomerular filtration rate <60 mL/min/m^2^), which is consistent with what was observed in the comprehensive Aotearoa New Zealand All Cardiology Services Quality Improvement nationwide cardiovascular database.^25^ From a biological plausibility perspective, patients with renal impairment are more likely to have calcific coronary disease and also more likely to have accelerated atherosclerosis, which may in part be related to the same traditional cardiovascular risk factors driving the concomitant kidney disease but equally may also be a manifestation of the impaired calcium-phosphate homeostasis, which occurs with progressive renal failure.^26^^,^^27^

With regard to using IVL in an off-label fashion in a newly deployed but underexpanded stent, although there is bench-top electron microscopy evidence suggesting disruption of the drug polymer on drug-eluting stents from the sonic pressure waves of the IVL device, in the same ex vivo study, the authors also demonstrated that the stent polymer becomes disrupted each time it gets pushed and pulled through a (simulated) calcified coronary artery.^28^ Furthermore, the investigators of the recently published ELLIS (Comparative Analysis of Excimer Laser Coronary Angioplasty and Intravascular Lithotripsy on Drug-Eluting Stent as Assessed by Scanning Electron Microscopy) study demonstrated minimal polymer damage using both IVL and saline-ELCA in comparison to contrast-ELCA and even high-pressure balloon dilatation, which caused significant damage to the polymer coating on Onyx Frontier stents (Medtronic). Of note was the absence of any alterations seen in the polymer-free Cre8 stents (CID S.p.A.) used in a comparator arm.^29^ Indeed, although our cohort only had 9 cases in which IVL was performed in a newly-implanted stent, it is worth noting that none of these patients had any adverse cardiac events at 12-month follow-up. Ultimately, in the absence of any long-term follow-up data on these patients, it remains to be seen whether IVL in a newly deployed stent has a significant impact on long-term clinical end points.

Our study has a number of limitations. First, it is a nonrandomized retrospective cohort study. In addition, the size of the group is small given that IVL is used infrequently in the setting of an underexpanded stent and as such, it is not possible to infer what patient or lesion-specific characteristics may help identify those patients who are more likely to respond to IVL in this setting. The rate of use of intravascular imaging in our cohort was also modest, and larger cohorts with higher rates of intravascular imaging may help to contribute to the understanding of which mechanisms of stent failure and configurations of in-stent and behind-stent coronary calcium are likely to be amenable to in-stent IVL.

## Conclusions

This multicenter retrospective analysis of IVL use in underexpanded stents demonstrates that IVL is both efficacious in the treatment of stent underexpansion due to coronary calcification as well as safe with low rates of procedural complications and a 12-month MACE rate comparable to that of PCI in de novo coronary disease. However, randomized studies are required to further elucidate the optimal approach for the treatment of stent underexpansion.
